# Experiences of exercise and mental well-being among psychiatric inpatients: a qualitative study

**DOI:** 10.3389/fpsyt.2026.1907931

**Published:** 2026-10-19

**Authors:** Martin Bystad, Anna Lodewijks, Sveinung Rydland, Gunn Pettersen, Lorena García-Fernández, Roberto Rodriguez-Jimenez, Rolf Wynn

**Affiliations:** 1 Division of Substance Use and Mental Health, University Hospital of North Norway, Tromsø, Norway; 2 Department of Psychology, UiT The Arctic University of Norway, Tromsø, Norway; 3 Department of Health and Care Sciences, UiT The Arctic University of Norway, Tromsø, Norway; 4 Department of Clinical Medicine, Universidad Miguel Hernández, Alicante, Spain; 5 CIBERSAM-ISCIII (Biomedical Research Networking Centre for Mental Health), Madrid, Spain; 6 Department of Legal Medicine, Psychiatry, and Pathology, Complutense University of Madrid (UCM), Madrid, Spain; 7 Health Research Institute Hospital 12 de Octubre (imas12), Madrid, Spain; 8 Department of Clinical Medicine, UiT The Arctic University of Norway, Tromsø, Norway

**Keywords:** anxiety, exercise, guidance, impact, inpatient, mental well-being, psychiatry, qualitative

## Abstract

**Background:**

Exercise can have a range of benefits for both mental and physical health. There is a lack of research on how inpatients in psychiatric care experience physical exercise, as most existing studies focus on outpatients. The aim of this study was to explore how psychiatric inpatients experience exercise, in particular with respect to their mental well-being.

**Methods:**

We conducted qualitative interviews with eight inpatients (five male, three female, mean age of 53.9) with serious mental health disorders who had participated in individualized exercise sessions led by an exercise physiologist during their hospital stay we used reflexive thematic analysis.

**Results:**

We identified four key themes: follow-up support, symptom reduction, sense of mastery, and activation during hospitalization. A particularly notable finding was that participants reported immediate improvements in mood and perceived mental well-being following exercise. The presence of an exercise physiologist was described as a crucial source of motivation and support.

**Conclusion:**

Overall, the study suggests that structured opportunities for exercise with individualized professional support may be a valuable component of psychiatric inpatient care.

## 1 Introduction

Numerous studies have found that physical exercise can play a significant role in improving mental health, and in reducing symptoms of depression, anxiety, and distress (1, 2). Several meta-analyses have found that exercise can be effective for patients with depression, and also for patients with anxiety (1, 3). One study suggests that high levels of cardiovascular fitness are associated with reduced consumption of anxiolytics and antidepressants (4). Both cardiovascular exercise and strength training exercise can be effective, especially for depression (5). A survey reported that 80% of the mental health professionals strongly agreed that exercise can be a significant contribution to preventing and treating mental health disorders (6).

Regular exercise also has a large number of long-term physical health benefits (7, 8).

Somatic conditions are prevalent among patients with poor mental health (9). For instance, patients with serious mental illness have a 12 year reduction in life expectancy, probably at least in part due to poor lifestyle (10).

A study found that healthy individuals who exercised regularly experienced 43% fewer days of poor mental health in the past month compared to those who did not exercise, even after accounting for physical and sociodemographic factors (11). Recent literature has also emphasized potential neurobiological, psychological, and self-efficacy-related pathways through which exercise may contribute to mental well-being (12). Exercise can contribute to better well-being through the reduction of stress hormones, enhancement of sleep quality, release of endorphins, relief of muscle tension, and can also improve overall physical health (12, 13). Exercise can foster a sense of achievement, raise self-esteem and reduce feelings of helplessness (14, 15). This is especially valuable for patients with mental health challenges. A previous study (16) revealed that 93% of psychiatric inpatients reported an alleviation of mood by following an exercise program. Despite all the evidence, exercise is still not widely applied as a treatment mode in the mental health care system in Norway and many other countries (1).

Research focusing specifically on physical exercise among psychiatric inpatients remains limited, although a growing body of evidence has addressed exercise within inpatient mental health settings (16–18). Previous evidence of improved mood following structured inpatient exercise has relied primarily on self-reported pre- and post-session outcomes (16). Rogers et al. (17), used qualitative interviews with patients in a medium-secure forensic setting. They identified perceived psychological benefits of exercise as well as barriers related to mental health symptoms, medication side effects, environmental restrictions, and staff support. However, their findings were based on two secure wards and excluded patients who were not well enough to participate in interviews. This limits the generalizability of the findings.

More recently, McKenna et al. (18) systematically reviewed 12 studies examining barriers to implementing physical exercise in inpatient mental health settings. The review identified barriers related to patients’ mental and physical health, service providers, the inpatient environment, and institutional culture. The included literature was heterogeneous in design and population, several studies focused exclusively on healthcare professionals, and most addressed physical exercise in general. McKenna et al. (18) emphasized the need for individual exercise programs and specialist professional roles within multidisciplinary inpatient care.

Although previous studies have identified important barriers to exercise in inpatient psychiatry and suggest potential psychological benefits, less is known about how psychiatric inpatients experience exercise. This is particularly relevant given recent calls for greater attention to exercise within inpatient mental health care (18). The present study therefore aimed to explore psychiatric inpatients’ experiences of exercise and its perceived impact on mental well-being during hospitalization.

## 2 Materials and methods

The study took place in the Division of Substance Use and Mental Health at the University Hospital of North Norway. The psychiatric clinic has two fully equipped gyms that inpatients may use, and two exercise physiologists offer support and follow-up to patients who are interested in physical exercise.

### 2.1 Study design

This was a qualitative study, based on a purposive sample of eight inpatients who had participated in individualized physical exercise sessions supported by exercise physiologists. The participants participated in open-ended interviews, and the transcriptions were analysed by drawing on principles from reflexive thematic analysis (19, 20).

### 2.2 Participants

Participants Eight patients admitted to the Division of Substance Use and Mental Health at the University Hospital of North Norway participated in individual interviews. The participants were recruited by an exercise physiologist (S.R.), who asked inpatients if they wanted to participate in a qualitative study. In order to participate in the study, we required that the patient had completed at least two supported exercise sessions. All participants were assessed as competent to provide informed consent.

The participants had a mean age of 53.9 (range from 31 to 84). Five were male and three were female. The participants suffered from schizoaffective disorder/schizophrenia (3), substance use disorder (3), and depressive disorder (2). Given the small and heterogeneous sample, subgroup analyses were not performed. However, we remained attentive to divergent experiences across participants.

At the time of the interviews, participants had been hospitalized for varying durations (approximately three to six weeks). All participants had engaged in individualized exercise sessions supervised by an exercise physiologist (S.R.), with variation in the number of completed sessions.

The exercise intervention was individualized and adapted according to patient preference, goals, physical training status and mental capacity. Despite this, all participants engaged in broadly similar types of exercise (i.e. a combination of resistance training and aerobic exercise during their inpatient stay). Participants’ training status varied from having regularly trained for several years, to having no previous experience with structured exercise, with the majority having very little experience with structured exercise. Exercise sessions typically lasted 45–60 minutes, conducted twice per week, although the overall frequency ranged from one to three sessions weekly. The total number of exercise sessions completed varied between participants; however, exact participant-level session counts were not available for the present analysis. A minimum of two sessions were needed to be eligible for participation in this study.

Resistance training typically involved whole-body exercises performed in two to three set with four to 15 repetitions following principles of gradual progression when tolerated. Participants were encouraged to increase either load or repetitions when reaching the upper end of the prescribed repetition range. For instance, an the weight lifted could be increased when a participant could perform 10 repetitions of an exercise.

Aerobic exercise included both interval-based and continuous training. This was performed on a treadmill and with minor adaptations (e.g., cycling) when needed or preferred. The exercise intervention relied on individual adaptation, safety, and feasibility within a psychiatric inpatient setting rather than strict standardization. Exercise intensity was individually adjusted according the participant’s goals, physical capacity, and prior experiences. A variety of methods were used to monitor exercise intensity, depending on the participants’ own preferences. Heart rate monitors were used for some (Polar H10), while others used the Borg scale, or a combination of the two.

### 2.3 Interviews

Two of the authors (M.B. and A.L.), who are clinical psychologists, conducted the interviews. They interviewed patients that they had no treatment responsibility for. The interview guide was semi-structured, with open-ended questions. In the interviews, the participants were asked individually why they chose to engage in exercise activities and how exercise affected their mental health.

Each participant took part in a telephone interview lasting approximately 30 minutes (range 20–60 minutes). The variation in interview duration likely reflects differences in the level of detail provided by participants. All interviews were audio-recorded and transcribed verbatim.

The participants were encouraged to give honest feedback, and informed that it would not affect their future treatment. The participants were encouraged to speak freely about their experiences. The primary research question was: How does exercise impact well-being and mental health during hospitalization? Follow-up questions in the interview guide included: ‘What are the main reasons you choose to exercise during your stay?’, ‘How do you feel exercise influences your mood?’, ‘Which effects do you think exercise has on your body?’, ‘What role does exercise play in your daily routine while you are hospitalized?’, ‘Sometimes exercise can feel challenging to complete because you might feel extra tired or down. How does it feel for you?’, ‘If you were to highlight three important points from our conversation today, what would they be?’

During data collection, we continuously assessed whether additional interviews contributed new insights. Given the focused study aim and the richness of the interview material, the sample was considered adequate. The final interviews did not yield substantially new codes or themes, suggesting sufficient thematic depth.

### 2.4 Analysis

This study employed reflexive thematic analysis as described by Clarke and Braun (19) to explore psychiatric patients’ experiences with physical exercise during inpatient treatment. We relied on a six-step approach, according to the thematic analysis model (20). We wanted to identify commonalities and differences in how the participants perceived and engaged with exercise as part of their mental health care.

The analysis was carried out with a contextualist perspective. We recognized participants’ accounts as meaningful descriptions of their experiences while also acknowledging that themes were developed through an interpretive process. We selected the method of reflexive thematic analysis because it allowed for the systematic identification and interpretation of shared patterns of meaning, while also keeping a focus on the context of the participants’ experiences.

First, we familiarized ourselves with the data by repeatedly reading the interview transcripts, noting initial impressions and reflections mainly related to the participants’ perceptions of exercise during hospitalization. Second, initial codes were systematically generated across the dataset by identifying meaningful features of the data that related to the experiences. Third, these codes were collated into potential themes that captured key aspects of the participants’ exercise experiences in the context of psychiatric treatment. Relevant data extracts were grouped under each theme to represent the various dimensions of exercise as reported by the participants. In the fourth step, we reviewed and improved the themes in two ways: first, by making sure they matched the coded parts of the data, and second, by checking that they covered the whole dataset. Next, we defined and named the themes to clearly reflect their content and significance within the broader narrative of exercise experiences during hospitalization. Finally, the analysis was written up in a clear report, using quotes from participants to illustrate each theme. The results were connected to the research questions and earlier studies on exercise and mental health, and discussed in light of implications for clinical practice.

Throughout the analysis, the authors engaged in collaborative discussions to critically reflect on interpretations and refine the themes developed from patterns of meaning within the dataset.

Reflexivity was actively considered throughout the entire study. The interviewers were clinical psychologists working within the same hospital, but without any treatment responsibility for the participants. The authors continuously reflected on how their professional backgrounds and preconceptions might influence the interpretation of the results.

### 2.5 Ethics

The project was approved by the Data Protection Officer of the University Hospital of North Norway and followed the principles of the Helsinki Declaration in terms of informed consent, the option of unconditional withdrawal and anonymization.

Quality assurance projects such as the present are not covered by the Health Research Act and are therefore exempt from ethical review board approval, according to Guidance from the National Research Ethics Committee (https://www.forskningsetikk.no/retningslinjer/med-helse/helseforskningslovens-saklige-virkeomrade/).

Quality assurance projects typically examine routine treatments in the clinical context that they are usually offered. This excludes procedures typical of many research projects, such as randomization, control groups, and experiments with previously unused treatments.

The participants received a gift certificate of 200 NOK (ca. 18 USD) for their participation. Participation was voluntary. Patients were approached by an exercise physiologist (S.R) and provided with information about the study (both orally and written). It was emphasized that participation or non-participation would not influence their treatment or relationship with staff. To ensure informed consent, the information was communicated in a clear and precise manner. All participants were given the opportunity to ask questions before consenting. Only patients assessed as having the capacity to consent were included. We remained attentive to participants’ mental state throughout the process to ensure ongoing voluntariness.

## 3 Results

The participants’ responses were divided into four main themes: 1) ‘Follow-up support’, 2) ‘Symptom reduction’, 3) ‘Sense of mastery’ and 4) ‘Activation during hospitalization’. These four themes will be described in more detail below:

The four themes represent distinct but related aspects of participants’ experiences of exercise. Follow-up support concerns the interpersonal and professional support that facilitated exercise, whereas symptom reduction reflects perceived changes in mood and emotional state. Sense of mastery captures experiences of accomplishment and confidence, while activation during hospitalization concerns the role of exercise in providing activity, structure, and meaning during the hospital stay.

### 3.1 Follow-up support

Support from the exercise physiologists was highlighted as a crucial factor for initiating and maintaining exercise. The participants described how a structured approach and individualized adaptation created a safe framework in which they felt cared for. One participant expressed it like this:

‘The exercise physiologist was supportive and tailored the training to my needs. We had a program, and I received follow-up. I was brought to training by the exercise physiologist regardless of how I was feeling, even when I was at my lowest. I have to praise the exercise physiologist. He was a great support, a good listener, and always calm.” (Participant 1, female).

This quote illustrates how important the participants felt it was to have a consistent contact person who not only tailored the exercise but also ensured continuous follow-up, even during periods of low motivation or poor condition. Another participant had a similar viewpoint and emphasized how vital the personal support was to get started with exercise:

“If I didn’t have this offer, I would’ve struggled much more to get started with exercising. The threshold would’ve been even higher. In this situation, it is crucial to have support from someone who can motivate you. I needed that help to get started.” (Participant 2, male).

For a third participant, this support acted as a kind of ‘kickstart’:

“It is indeed difficult to start an exercise program when you are very depressed and out of shape. I felt he (the exercise physiologist) gave me a start, I would call it a “kickstart.” (Participant 3, male).

Together, these quotes highlight that support from the exercise physiologists was not just about the exercise itself, but also about providing safety, motivation, and continuity that helped overcome the barriers to activity.

Participants also described barriers to initiating or maintaining exercise. Low mood, fatigue, and physical discomfort could make it difficult to get started or participate. This was particularly evident during periods of depression:

“When I’m depressed, it’s difficult to have the energy to exercise. It’s so difficult to get started.” (Participant 5, male).

For some participants, professional support was therefore particularly important in helping them initiate exercise despite these barriers.

### 3.2 Symptom reduction

The participants stated that they experienced that exercising led to improvements in mood and emotional balance. Many described how exercise instantly helped reduce anxiety, anger, and depressive symptoms. One participant said:

“I noticed that my mood improved immediately. You get a victory for yourself. It’s like, ‘Yes, I did it!’ feeling.” (Participant 1, female).

This expresses the positive sense of accomplishment that comes with physical activity — a feeling of having achieved something concrete, which boosts self-esteem and joy. Another participant described exercise as a form of medicine:

“Exercise is my greatest medicine. Yesterday, I felt upset and anxious. But after an exercise session I was calm and collected.” (Participant 4, female).

Another participant elaborated on how he perceived the effect of exercise to last beyond the immediate moment:

“Exercise improves my mood, not just temporarily but for the rest of that day. I get that feeling of being relaxed after an exercise session.” (Participant 8, male).

A similar perspective came from a participant who noticed that exercise especially helped with anger and difficult emotions:

“What I notice the most is that exercise helps with anger and difficult emotions. It helps me cope with being hospitalized. After an exercise session I can be very sore and tired in my body. But that’s how it should be. I am proud of myself after hard sessions with exercise.” (Participant 4, female).

Here, the participant expresses that exercise not only impacts her emotional state, but also brings a sense of pride and mastery. Another participant summed it up as:

“I feel like a new human being after each exercise session!” (Participant 3, male).

This quote illustrates the transformative experience many participants had, where exercise gave them renewed energy and improved quality of life. However, the perceived effects were not uniformly strong. Some participants were uncertain whether exercise affected specific symptoms such as sleep or energy, and one participant reported little noticeable change in some areas. Another emphasized that exercise did not remove depressive thoughts, but rather helped make difficult days more manageable:

“It’s not like I suddenly become super happy. I still have just as many heavy thoughts and feel depressed. But it helps you get through the day.” (Participant 4, female).

### 3.3 Sense of mastery

Exercise gave the participants a feeling of mastery that often extended beyond the exercise session itself and influenced other areas of their lives. This feeling contributed to increased self-confidence and motivation:

“Exercise made me gradually achieve more in other areas too. It was a victory for myself.” (Participant 2, male).

For many, exercise became a catalyst for positive changes in everyday life. Another participant described the experience of mastery as follows:

“You get a sense of mastery when you’ve exercised, and it goes well. It feels good to have exercised, and in the long run, I think it”s very good for mental health.’ (Participant 5, male).

A third participant emphasized the importance of both autonomy and mastery in the exercise experience:

“Everybody wants to be able to handle things themselves. I noticed that my sense of mastery got better with exercise. It makes me more confident to know that exercise helps.” (Participant 8, male).

This quote illustrates how some participants felt exercise could boost their ability to cope, which is essential for psychological wellbeing.

### 3.4 Activation during hospitalization

For many participants, exercise was a key activity that helped structure their days and reduce feelings of isolation and passivity during hospitalization:

“It made it easier to be hospitalized. I had something to look forward to and something to do instead of just sitting in the ward.” (Participant 1, female).

This quote illustrates how physical activity can create meaning and help break up the often monotonous and passive hospital days. Another participant described how exercise helped her cope with the hospitalization:

“I feel calmer when I’ve exercised. If I don’t exercise, I just pace back and forth in my room.” (Participant 4, female).

This highlights exercise as an important coping strategy to reduce restlessness and stress in a difficult situation. A third participant stressed how central exercise was to maintaining a sense of normalcy and control:

“Exercise means a lot, and it’s a coping strategy. It keeps the days going. It’s tough because you’re dealing with a lot of issues and problems. If I hadn’t been able to train, I don’t know what would’ve happened”. (Participant 6, female).

Here, exercise is described as a vital activity helping the participant get through tough periods.

## 4 Discussion

### 4.1 General discussion

The aim of this study was to explore psychiatric inpatients’ experiences of exercise and its perceived impact on mental well-being during hospitalization. Overall, participants described exercise as having a positive role in their perceived mental state. These findings reflect participants’ subjective experiences and should not be interpreted as evidence of treatment efficacy or causal effects of exercise.

An overall finding was that exercise can play an important role in improving how inpatients perceive their mental state. A central finding was that the participants underlined the importance of the support and follow-up that they received from the exercise physiologist. We found that the participants emphasized the challenges with performing exercise sessions on their own, without any support, which has also been noted in a prior study (17). Previous studies have found that lack of motivation for exercise was a main barrier for psychiatric inpatients (6, 18). It may be unrealistic to expect hospitalized patients to be able to exercise entirely on their own without the guidance of qualified staff, e.g., an exercise physiologist or physiotherapist.

The importance that our participants placed on support and follow-up from the exercise physiologist shows that structural and relational factors are central to the success of the intervention. A heavy symptom burden and low motivation coupled with a restrictive inpatient environment are important barriers to physical exercise for inpatients (6, 18). In our study, the exercise physiologist helped the participants overcome these barriers by offering emotional and practical support, professional knowledge, safety and predictability. Our results suggest that adequate support and follow-up are key to successfully implementing physical exercise interventions for inpatients in psychiatric care.

Furthermore, participants reported perceived acute reductions in symptoms, including improvements in mood following exercise sessions. This is consistent with previous studies reporting affective responses to exercise (21, 22). However, the present qualitative design does not allow us to determine whether these perceived changes were caused by exercise itself. Rather, the findings demonstrate that participants experienced and attributed positive changes in their emotional state to the exercise sessions.

The immediate perceived improvements described by participants may reflect both biochemical and psychological processes associated with exercise. Engaging in exercise is associated with a range of neurobiological and psychological responses that may contribute to changes in mood and perceived well-being (23). Exercise can also foster a sense of accomplishment and mastery, and may also redirect attention away from negative thoughts and provide a break from daily stressors (14). While medications are passive treatments managed by healthcare providers, exercise is something patients can do independently. This can strengthen a sense of autonomy and agency in their recovery.

Our findings may also be understood within a behavioral activation framework. Behavioral activation proposes that withdrawal and inactivity may reduce access to positively reinforcing experiences, whereas engagement in meaningful activities may improve mood and functioning (13, 24). From this perspective, exercise may interrupt inactivity and provide reinforcing experiences through improved mood, daily structure, and a sense of accomplishment and mastery. Professional support may facilitate this activation when symptoms, fatigue, or low motivation make initiating exercise difficult. The immediate positive consequences of exercise may, in turn, reinforce continued engagement in physical activity. Thus, behavioral activation provides a possible functional explanation for some of the improvements described by the participants. However, as behavioral mechanisms were not directly examined, this interpretation remains theoretical.

Importantly, the perceived improvements cannot necessarily be explained by exercise alone. Individualized professional support, social interaction, increased structure and meaningful activity, temporary removal from the ward environment and behavioral activation may also have contributed to participants’ experiences. As the study did not include a comparison condition, their relative contributions cannot be determined. Thus, the findings should be understood as reflecting participants’ experiences of professionally supported exercise rather than effects specific to exercise itself.

A loss of activities, a loss of well-being, and boredom are challenges for many patients (25). It is a noteworthy finding that exercise made their hospital stay more manageable. For inpatients, exercise can give structure to a daily routine, create opportunities for social interaction, and promote a sense of normalcy. For inpatients, enjoyable activities can be important for their well-being and recovery. When psychiatric inpatients become bored this can negatively affect their experiences with treatment (26).

In our study, we found that exercise provided a sense of mastery, a finding that is supported by prior research (27, 28). A sense of mastery leads to reduced stress and greater well-being (27, 28). Thus, the positive experience of exercise among our participants may partly be explained by sense of mastery. According to Bandura’s self-efficacy theory (29–31), experiencing mastery will enhance an individual’s beliefs in their ability to cope with difficulties and in influencing outcomes. Thus, inpatients completing physical exercise in spite of their challenges may experience an increased sense of confidence and coping that might be generalized beyond physical exercise.

For some patients, exercise can lead to an unhealthy focus on body image, weight, and performance (32). For such patients, exercise can evolve into a compulsive behavior, where the patient feels driven to exercise even when their body demands rest (33). None of our participants reported such experiences. However, it is crucial that psychiatric inpatients are assessed for their suitability for using exercise as a treatment.

It is still unclear which types of exercise and levels of intensity are most effective for improving mental health. Some studies have found that high-intensity exercise can be the most effective approach, at least for depression (3). However, all types of physical activity, whether light, moderate, or vigorous, have been demonstrated to offer long-term protection against psychological distress (34). Exercise can serve as a treatment in addition to medication and psychotherapy during hospitalization, but it is typically not intended to replace standard treatment. Physical activity should therefore be viewed as a supplement to established interventions for the treatment of mental illness.

### 4.2 Clinical implications

The study has important clinical implications. The results may suggest that more inpatients should be given the opportunity to participate in regular physical exercise. In line with prior research, our findings suggest that many patients will have a need for support and facilitation, in order to overcome motivational challenges (6, 18). Some participants described that they felt exercising had a rapid positive effect on their mental state, as previously observed (21, 22). The reported improved sense of mastery supports former research connecting physical exercise, well-being and self-efficacy (14, 27). However, the present qualitative study was not designed to examine treatment efficacy or optimal exercise dose, or to make comparisons with other interventions.

Future research on the importance of physical exercise in psychiatric treatment should place greater emphasis on psychiatric inpatients, a group that remains significantly underrepresented in existing exercise and mental health studies. Investigating how exercise interventions can be tailored to specific psychiatric disorders and individual needs within this population is especially important. Future studies should aim to include a broader and more diverse sample to capture a wider range of experiences.

Moreover, exploring how different forms of exercise, such as yoga, strength training, or group-based activities, affect various subgroups of inpatients could yield valuable insights. Research should also examine strategies for overcoming common barriers to participation, including low motivation, fatigue, and physical limitations, challenges that are particularly relevant in inpatient settings.

The present study shows how physical exercise was experienced by patients currently in inpatient treatment, i.e., patients that were in a phase of worsening of their illness, typically with more severe symptoms. These patients also experienced the restrictions that follow from inpatient status, and had limited activities to choose from. The study finds that the patients describe their participation in physical activity as beneficial for their health, motivating and meaningful, underlining the need to more systematically give inpatients the opportunity to exercise. The study also suggests that it is central to give adequate support and follow-up to secure a positive exercise experience for participants.

### 4.3 Strengths and limitations

The present study is one of the first to explore how inpatients in psychiatry experience guided physical exercise sessions. Drawing on a qualitative analysis, we find that several participants experienced an immediate positive impact of the sessions on their mental state, and that structured professional support may be important to this experience.

However, the present study has some limitations. While qualitative interview-based studies such as the present typically have few participants, the sample size of eight participants is relatively small. To be admitted to inpatient psychiatric care, individuals must typically meet criteria for a serious mental health disorder. This criterion inherently limits the pool of eligible participants. Consequently, this has affected our ability to recruit a sufficiently large and diverse sample, thereby limiting the breadth of experiences represented in the study. Caution is required when considering the transferability of our findings to other settings or populations. Recruiting inpatients in psychiatric settings can be particularly difficult due to high symptom severity, including acute distress and fluctuations in mental state. Additional barriers include the involuntary nature of some admissions (35), concerns about confidentiality, and general mistrust toward research. Practical challenges (e.g. short lengths of stay, limited staff availability, and ethical considerations around obtaining informed consent) can further hinder recruitment efforts in these settings. A second limitation is a possible selection bias, as those who chose to participate may have been more positively inclined toward exercise. Furthermore, recruitment was carried out by an exercise physiologist who provided professional guidance during exercise sessions. This may have introduced selection and social desirability bias, as patients with more positive experiences could have been more willing to participate or to offer a positive evaluation. The results may not fully represent the experiences of all psychiatric inpatients.

Other limitations should also be considered. The heterogeneity of psychiatric diagnoses and variation in exercise exposure may have influenced participants’ experiences. As exercise was provided within ongoing inpatient treatment and the study had no comparator, it is difficult to distinguish the perceived effects of exercise from those of other aspects of inpatient treatment.

The timing of interviews relative to exercise sessions may also have influenced participants’ accounts. Finally, telephone-based interviews limited access to non-verbal cues, and the single-center Norwegian setting may limit the transferability of the findings to other inpatient settings.

Future research should not only explore how exercise can be adapted to engage a broader and more diverse group of patients, but also place greater focus on psychiatric inpatients specifically. This population remains underrepresented in current research, despite being in a particularly vulnerable phase of illness.

## 5 Conclusions

This study describes how a group of inpatients perceived individualized, professionally supported exercise during their hospital stays. The participants expressed perceived improvements in their mood and mastery, and reported that the exercise led to greater activation and structure. The individualized follow-up by the exercise physiologist was described as especially important for initiating and keeping up exercise. Our findings underline the potential value of helping inpatients to engage in physical activity. Further research is needed to examine efficacy, identify active components of the exercise intervention, and understand how exercise can be effectively integrated into inpatient treatment and sustained beyond discharge.

## Data Availability

The datasets presented in this article are not readily available because the transcripts on which the study is based are unavailable due to privacy restrictions. Requests to access the datasets should be directed to Martin.Kragnes.Bystad@unn.no.

## References

[1] Singh B Olds T Curtis R Dumuid D Virgara R Watson A . Effectiveness of physical activity interventions for improving depression, anxiety and distress: an overview of systematic reviews. Br J Sports Med. (2023) 57:1203–9. doi: 10.1136/bjsports-2022-106195 36796860 PMC10579187

[2] Pearce M Garcia L Abbas A Strain T Schuch FB Golubic R . Association between physical activity and risk of depression: a systematic review and meta-analysis. JAMA Psychiatry. (2022) 79. doi: 10.3389/fnagi.2021.815439 35416941 PMC9008579

[3] Noetel M Sanders T Gallardo-Gómez D Taylor P Del Pozo Cruz B van den Hoek D . Effect of exercise for depression: systematic review and network meta-analysis of randomised controlled trials. BMJ. (2024) 384:e075847. doi: 10.1136/bmj-2023-075847 38355154 PMC10870815

[4] Havnen A Zotcheva E Bjerkeset O Sui X Ernstsen L . Cardiorespiratory fitness and incident use of anxiolytics and antidepressants in adults. A linkage study between HUNT and the Norwegian Prescription Database. J Affect Disord. (2023) 339:111–7. doi: 10.1016/j.jad.2023.07.029 37437717

[5] Heissel A Heinen D Brokmeier LL Skarabis N Kangas M Vancampfort D . Exercise as medicine for depressive symptoms? A systematic review and meta-analysis with meta-regression. Br J Sports Med. (2023) 57:1049–57. doi: 10.1136/bjsports-2022-106282 36731907 PMC10423472

[6] Imboden C Mikutta C Hemmeter U Seifritz E Claussen MC . Physical activity in psychiatry: Current state and challenges: An online-survey on sports psychiatry in health professionals. Sports Psychiatry. (2024) 3:187–95. doi: 10.1024/2674-0052/a00006930224840

[7] Reiner M Niermann C Jekauc D Woll A . Long-term health benefits of physical activity--a systematic review of longitudinal studies. BMC Public Health. (2013) 13:813. doi: 10.1186/1471-2458-13-813 24010994 PMC3847225

[8] Warburton DER Bredin SSD . Reflections on physical activity and health: What should we recommend? Can J Cardiol. (2016) 32:495–504. doi: 10.1016/j.cjca.2016.01.024 26995692

[9] Hassan S Byravan S Al-Zubaidi H . Improving physical healthcare provided to psychiatric inpatients at an acute mental health trust. BMJ Open Qual. (2019) 8:e000537. doi: 10.1136/bmjoq-2018-000537 31523731 PMC6711444

[10] Chang CK Hayes RD Perera G Broadbent MT Fernandes AC Lee WE . Life expectancy at birth for people with serious mental illness and other major disorders from a secondary mental health care case register in London. PloS One. (2011) 6:e19590. doi: 10.1371/journal.pone.0019590 21611123 PMC3097201

[11] Chekroud SR Gueorguieva R Zheutlin AB Paulus M Krumholz HM Krystal JH . Association between physical exercise and mental health in 1·2 million individuals in the USA between 2011 and 2015: a cross-sectional study. Lancet Psychiatry. (2018) 5:739–46. doi: 10.1016/s2215-0366(18)30227-x 30099000

[12] Kanani J Baldania S Nasif D . Integrating physical therapy and exercise in mental health: A novel framework for suicide prevention and depression management. J Neurosci Neurol Surg. (2025) 17. doi: 10.31579/2578-8868/35942704289

[13] Smith PJ Merwin RM . The role of exercise in management of mental health disorders: An integrative review. Annu Rev Med. (2021) 72:45–62. doi: 10.1146/annurev-med-060619-022943 33256493 PMC8020774

[14] Ciccolo JT Whitworth JW Nosrat S . Psychological benefits of exercise. In: APA Handbook of Sport and Exercise Psychology: Exercise Psychology, vol. 2. American Psychological Association, Washington, DC, US (2019). p. 93–108.

[15] Mahindru A Patil P Agrawal V . Role of physical activity on mental health and well-being: A review. Cureus. (2023) 15:e33475. doi: 10.7759/cureus.33475 36756008 PMC9902068

[16] Tomasi D Gates S Reyns E . Positive patient response to a structured exercise program delivered in inpatient psychiatry. Global Adv Health Med. (2019) 8:2164956119848657. doi: 10.1177/2164956119848657 31192053 PMC6540485

[17] Rogers E Papathomas A Kinnafick FE . Inpatient perspectives on physical activity in a secure mental health setting. Psychol Sport Exercise. (2021) 52:101827. doi: 10.1016/j.psychsport.2020.10182742574925

[18] McKenna C Moyo B Goodwin J . Barriers to using physical exercise as an intervention within inpatient mental health settings: A systematic review. Int J Ment Health Nurs. (2024) 33:817–33. doi: 10.1111/inm.13302 38332557

[19] Clarke V Braun V . Thematic analysis. In: Encyclopedia of Critical Psychology. New York, NY: Springer (2014). p. 1947–52.

[20] Braun V Clarke V . Using thematic analysis in psychology. Qual Res Psychol. (2006) 3:77–101. doi: 10.1191/1478088706qp063oa

[21] Ensari I Greenlee TA Motl RW Petruzzello SJ . Meta-analysis of acute exercise effects on state anxiety: An update of randomized controlled trials over the past 25 years. Depression Anxiety. (2015) 32:624–34. doi: 10.1002/da.22370 25899389

[22] Bourke M Patten RK Klamert L Klepac B Dash S Pascoe MC . The acute affective response to physical activity in people with depression: A meta-analysis. J Affect Disord. (2022) 311:353–63. doi: 10.1016/j.jad.2022.05.089 35605707

[23] Matta Mello Portugal E Cevada T Sobral Monteiro-Junior R Teixeira Guimarães T da Cruz Rubini E Lattari E . Neuroscience of exercise: from neurobiology mechanisms to mental health. Neuropsychobiology. (2013) 68:1–14. doi: 10.1159/000350946 23774826

[24] Dimidjian S Barrera M Jr. Martell C Muñoz RF Lewinsohn PM . The origins and current status of behavioral activation treatments for depression. Annu Rev Clin Psychol. (2011) 7:1–38. doi: 10.1146/annurev-clinpsy-032210-104535 21275642

[25] Hoxmark E Wynn TN Wynn R . Loss of activities and its effect on the well-being of substance abusers. Scand J Occup Ther. (2012) 19:78–83. doi: 10.3109/11038128.2011.552120 21299367

[26] Chevalier A Ntala E Fung C Priebe S Bird VJ . Exploring the initial experience of hospitalisation to an acute psychiatric ward. PloS One. (2018) 13:e0203457. doi: 10.1371/journal.pone.0203457 30180196 PMC6122813

[27] Novic AJ Seib C Burton NW . Mastery, physical activity and psychological distress in mid-aged adults. Aust J Psychol. (2023) 75:2153623. doi: 10.1080/00049530.2022.2153623 40666003 PMC12175604

[28] Sargent-Cox KA Butterworth P Anstey KJ . Role of physical activity in the relationship between mastery and functional health. Gerontologist. (2015) 55:120–31. doi: 10.1093/geront/gnu042 24847845 PMC5994882

[29] Bandura A . Self-Efficacy: The Exercise of Control. W. H. Freeman. New York, NY. (1997).

[30] Ramsdal GH Wynn R . Do two weeks in a learning camp after ninth grade make a difference? Experiences of de-motivated boys with an increased risk of school dropout. Behav Sci. (2024) 14:189. doi: 10.3390/bs14030189 38540492 PMC10967892

[31] Hu Y Liu B . The impact of physical exercise on college students’ mental health through emotion regulation and self-efficacy. Sci Rep. (2025) 15:33548. doi: 10.1038/s41598-025-18352-9 41022943 PMC12480569

[32] Mathisen TF Bratland-Sanda S Rosenvinge JH Friborg O Pettersen G Vrabel KA . Treatment effects on compulsive exercise and physical activity in eating disorders. J Eating Disord. (2018) 6:43. doi: 10.1186/s40337-018-0215-1 30559966 PMC6293524

[33] Meyer C Taranis L Goodwin H Haycraft E . Compulsive exercise and eating disorders. Eur Eating Disord Rev. (2011) 19:174–89. doi: 10.1002/erv.1122 21584911

[34] Sheikh MA Vancampfort D Stubbs B . Leisure time physical activity and future psychological distress: A thirteen year longitudinal population-based study. J Psychiatr Res. (2018) 101:50–6. doi: 10.1016/j.jpsychires.2018.02.025 29550608

[35] Wynn R . Coercion in psychiatric care: clinical, legal, and ethical controversies. Int J Psychiatry Clin Pract. (2006) 10:247–51. doi: 10.1080/13651500600650026 24941142

